# Effect of Cholesterol Molecules on Aβ1-42 Wild-Type and Mutants Trimers

**DOI:** 10.3390/molecules27041395

**Published:** 2022-02-18

**Authors:** Trung Hai Nguyen, Phuong H. Nguyen, Son Tung Ngo, Philippe Derreumaux

**Affiliations:** 1 Laboratory of Theoretical and Computational Biophysics, Ton Duc Thang University, Ho Chi Minh City, Vietnam; nguyentrunghai@tdtu.edu.vn (T.H.N.); ngosontung@tdtu.edu.vn (S.T.N.); 2 Faculty of Applied Sciences, Ton Duc Thang University, Ho Chi Minh City, Vietnam; 3 Laboratoire de Biochimie Théorique, UPR 9080 CNRS, Université de Paris, 13 rue Pierre et Marie Curie, 75005 Paris, France; phuong.nguyen@ibpc.fr; 4 Institut de Biologie Physico-Chimique, Fondation Edmond de Rothschild, PSL Research University, 75005 Paris, France; 5 Institut Universitaire de France (IUF), 75005 Paris, France

**Keywords:** aggregation, amyloid-beta, mutants, cholesterol, simulations

## Abstract

Alzheimer’s disease displays aggregates of the amyloid-beta (Aβ) peptide in the brain, and there is increasing evidence that cholesterol may contribute to the pathogenesis of the disease. Though many experimental and theoretical studies have focused on the interactions of Aβ oligomers with membrane models containing cholesterol, an understanding of the effect of free cholesterol on small Aβ42 oligomers is not fully established. To address this question, we report on replica exchange with a solute tempering simulation of an Aβ42 trimer with cholesterol and compare it with a previous replica exchange molecular dynamics simulation. We show that the binding hot spots of cholesterol are rather complex, involving hydrophobic residues L17–F20 and L30–M35 with a non-negligible contribution of loop residues D22–K28 and N-terminus residues. We also examine the effects of cholesterol on the trimers of the disease-causing A21G and disease-protective A2T mutations by molecular dynamics simulations. We show that these two mutations moderately impact cholesterol-binding modes. In our REST2 simulations, we find that cholesterol is rarely inserted into aggregates but rather attached as dimers and trimers at the surface of Aβ42 oligomers. We propose that cholesterol acts as a glue to speed up the formation of larger aggregates; this provides a mechanistic link between cholesterol and Alzheimer’s disease.

## 1. Introduction

The two hallmarks of Alzheimer’s disease (AD) are extracellular amyloid-beta (Aβ) plaques of Aβ42 and Aβ40 peptides, the 42-residue species being the most toxic and intracellular neurofibrillary tangles built from hyperphosphorylated tau protein [1]. Despite extensive research, all drugs targeting Aβ and tau oligomers have failed in AD [2,3]. Among many cellular factors contributing to AD development, cholesterol plays a critical role via different actions. 

First, cholesterol is present in micro-dissected AD senile plaques with a molar ratio of 1:1 [4] and purified AD paired helical fragments of tangles [5]. The level of plasma cholesterol is 10% higher in AD patients than in normal individuals [6]. Cholesterol levels in the brain positively correlate with the severity of dementia in AD patients [7] in contrast to Aβ plaque burden, which correlates weakly with disease severity [8].

Second, cholesterol impacts Aβ production through the cleavage of the amyloid protein precursor (APP) [9]. Based on nuclear magnetic resonance and electron paramagnetic resonance, E22-N27 residues in the bulk solution and membrane-buried residues G29-G33 (using Aβ42 amino acid numbering) play a key role in cholesterol binding to APP [10]. Recently, it was proposed from atomistic molecular dynamics (MD) simulations that cholesterol modulates the conformation and activity of the C-terminal domain of APP directly through hydrogen bonding and indirectly through induction of the liquid-ordered phase [11]. Furthermore, Aβ accumulation in neurons is tightly regulated by cholesterol production in astrocytes [12], and elevated membrane cholesterol alters lysosomal degradation to induce Aβ degradation [13]. Finally, the apolipoprotein E gene, a major transporter of cholesterol in the brain, and in particular its e4 allele, the most genetic risk factor for AD, is associated with higher total cholesterol [14].

Third, numerous in vitro experiments have explored Aβ aggregation in a water– phospholipid–cholesterol membrane environment. They showed that cholesterol favors the formation of Aβ pores in the membrane of brain cells [15]. Cholesterol inserted into membranes facilitates aggregation of Aβ at the surface of the membranes at physiological concentrations, as reported by atomic force microscopy images [16]. Lipid membranes containing cholesterol also enhance the primary nucleation rate of Aβ42 aggregation by up to 20-fold, as reported by aggregation kinetics experiments [17].

At the theoretical level, many all-atom MD simulations were performed to study the interactions between Aβ42 peptides and membranes with various contents of cholesterol, revealing the mechanisms by which cholesterol changes the mechanisms of Aβ monomers and dimers binding and affinity to the lipid bilayer [16,18,19,20]. The binding of Aβ42 amyloid fibrils to the lipid bilayer was also investigated using coarse-grained MD simulations and showed that the addition of cholesterol increases the binding frequency and alters the binding interface and contacts [21].

Little is known about the interactions of Aβ42 peptides with free cholesterol in the bulk solution. The effect of unmodified cholesterol and charged cholesterol derivatives on Aβ40 fibril formation was explored using a Thioflavin T kinetic aggregation assay, atomic force microscopy and dynamic light scattering. At a concentration lower than the critical micelle concentration, and therefore assuming that cholesterol exists mainly as monomeric molecules, unmodified cholesterol and positively and negatively charged cholesterol derivatives accelerate the aggregation rate of Aβ40, the aggregation half time being reduced by 15 to 25% compared to Aβ40 peptide in phosphate buffer [22].

Using transmission electron microscopy, Harris et al. demonstrated the binding of soluble 10 nm diameter cholesterol-PEG 600 micelles to Aβ42 fibrils and proposed that binding involves the central hydrophobic core CHC spanning residues L17–A21 [23]. Atomistic 20 ns MD simulation of a system of four Aβ42-12 cholesterol molecules in a box of water showed stable contact between the benzyl group of F19 and the cholesterol micelle, which forms a flat surface and, notably, the steroid group of cholesterol [24]. It was suggested that the carboxyl terminus spanning residues have a higher tendency to interact with cholesterol and form β-sheet conformation [24]. 

It is well-established that lipids can be removed from the membrane because of oligomers–membrane interactions [25,26,27]. Recent experimental studies indicate the critical role of free phospholipids at a nanomolar to micromolar concentration in equilibrium with the membrane (large unilamellar vesicles) in forming Aβ–lipids complexes that favor amyloid–membrane poration [28,29]. We recently performed atomistic replica exchange molecular dynamics (REMD) simulations of an Aβ42 dimer and trimer with cholesterol using the protein AMBER99sb-ildn force field (also called AMBER ff99sb-ildn) with the TIP3P water force field. We reported on the drastic effect of cholesterol on the conformational ensemble of both Aβ42 species and showed multiple transient binding modes involving the residues L17–A21 and L30–M35 [30].

In this study, we further studied the impact of cholesterol with a ratio of 1:1 on Aβ42 trimers in the bulk solution. First, replica exchange with solute tempering (REST2) was chosen for sampling, as this procedure converges faster to equilibrium than REMD while requiring a smaller number of replicas [31,32,33]. Second, we used CHARMM36m, as this force field is more relevant for intrinsically disordered proteins [34] and small oligomers of Aβ [35,36,37,38,39]. This allows comparison with our previous REMD study using AMBER99sb-ildn TIP3P [30]. Third, we examined the impact of the A2T mutation, known to be AD protective, and the disease-causing A21G mutation [3] on the interactions of the Aβ trimer–cholesterol by MD simulations. It is to be noted that the A21G and A2T Aβ42 oligomers were extensively studied by experimental and theoretical means in the bulk solution [40,41,42,43,44,45,46]; however, no study reports on their interactions with free cholesterol.

## 2. Materials and Methods

As in our previous REMD study [30], the cholesterol molecule was geometrically optimized by quantum mechanics with the B3LYP functional and the 6-31G(d,p) basis set, and the cholesterol force field was parameterized using the general amber force field (GAFF) [42], the restrained electrostatic potential (RESP) method [47] and the B3LYP functional and the 6-31G(d,p) basis set [48].

In all simulations, Aβ42 peptides at pH 7 have NH3^+^ and CO2^−^ termini, deprotonated Glu and Asp, protonated Arg and Lys and neutral His with a protonated N_ε_ atom. The Aβ42/cholesterol system was neutralized by sodium ions. Prior to MD and REST2 simulations, all systems were minimized with harmonic restraints on the positions of the peptide Cα atoms and then equilibrated by MD at 310 K for 1 ns in the NVT ensemble, followed by 2 ns in the NPT ensemble.

**Figure 1:**
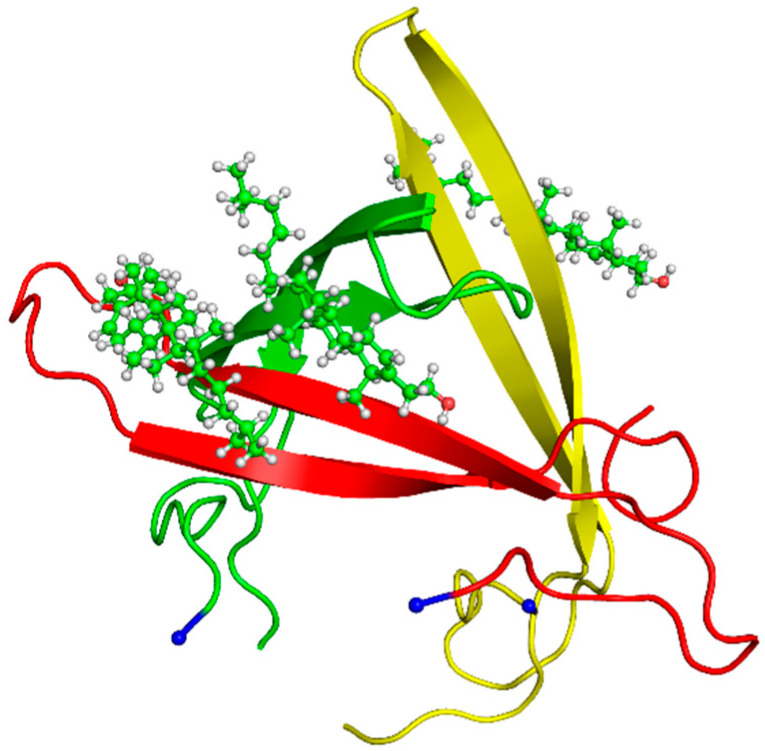
Initial REST2 conformation of Aβ42 trimer—three cholesterols with Aβ42 forming β-sheets at residues 13–21 and 29–35 of chain A (red), residues 16–22 and 31–37 of chain B (green) and residues 13–21 and 27–35 of chain C (yellow). Each N-terminus is shown by a blue ball. The three all-atom cholesterol molecules are shown in green.

In REST2, we used the same initial structure of the Aβ42 trimer–cholesterol system as in our REMD simulation inserted into a cubic water box of 7.26 nm size and 382.66 nm^3^ volume (10,869 water molecules). This structure with a high β-hairpin propensity of each chain was already discussed in many simulations of Aβ monomers [3,22] and oligomers [28,37,38,49,50] and in exploring amyloid oligomers with a peptide model system [51]. The equilibrated structure shown in Figure 1 started from three separated β-hairpins with one hairpin perpendicular to the other two peptides, and three cholesterol molecules randomly positioned and orientated with respect to Aβ42 peptides with a minimal distance of 1 nm from all Aβ42 atoms. 

REST2 simulation with the CHARMM36m-TIP3P force field was performed using NAMD [52]. We used a time step of 2 fs, a cutoff of 1.2 nm for Van der Waals interactions and a cutoff of 1.1 nm for electrostatic interactions using the particle mesh Ewald (PME) method [53]. REST2 scales the solute interactions by λ with the solute consisting of Aβ42 and cholesterol molecules, scales the solute–water interactions by λ^1/2^ and leaves the water–water interactions unaltered. Using T _min_ = 310 K and T _max_ = 500 K with the number of replicas set to 16, we performed REST2 simulation in the NPT ensemble at the temperature of 310 K for 250 ns on 16 replicas exchanging the following solute–solute corresponding temperatures of 310, 320, 330.4, 341.1, 352.1, 363.5, 375.3, 387.5, 400, 413, 426, 440.1, 454.4, 469.1, 484.3 and 500 K, i.e., for λ varying between 1 and 0.75. Exchanges of configurations between neighboring replicas were attempted every 1 ps. In the REST2 simulation, we used Nose–Hoover Langevin pressure control and Langevin temperature control as described in NAMD. 

Note that MD simulations of the selected WT, A2T and A21G systems were performed at 310 K using GROMACS [54] with the velocity-rescaling thermostat [55], a cutoff of 1.2 nm for Van der Waals interactions and a cutoff of 1.1 nm for electrostatic interactions using the PME method. 

To determine the interface between Aβ42 chains and cholesterols, we calculated the distances between heavy atoms of cholesterols and non-hydrogen atoms of the side chain residues of each Aβ42 chain. We considered that a contact formed between a side chain residue and cholesterol if there was at least one distance below 0.45 nm. For clarity, we report on the probability of side-chain contacts per Aβ42 chain and cholesterol molecule. We also calculated the percentage of cholesterol monomers, dimers+monomer and trimers. Monomers of cholesterol exist if there are no contacts between any two cholesterols, and trimers of cholesterol are formed if there is at least one contact between all cholesterols, a contact being defined if the intermolecular distance between any two heavy atoms is less than 0.45 nm. The DSSP protocol was used to determine the secondary structure of Aβ42 peptides [56].

The full energy landscape was approximated by a 2D energy landscape. First, the trajectory was projected on the first two principal components obtained after the diagonalization of the positional fluctuation covariance matrix of the backbone peptides and cholesterol atoms. Then, the free energy landscape was constructed from the previous data using the formula −RT × log(H(x,y)), where H(x,y) is the histogram of the two selected order parameters x and y [57]. The population of each minimum was determined by counting all conformations around each minimum. The representative structure or center of each cluster was obtained by the Daura clustering method [58].

In what follows, we used for comparison the time intervals 50–250 ns of the present REST2 simulation at 310 K and our previous REMD simulation at 315 K. Note that if death is imminent for humans at 315 K, atomistic protein simulations at 310 and 315 K led to very similar thermodynamic and structural properties. Additionally, a pure cholesterol bilayer was already explored at 310 K by all-atom MD simulations [59], and REST2 was successfully shown to simulate the weak binding of Aβ40 peptides on a lipid bilayer [32] and the lateral equilibration in mixed cholesterol-DPPC bilayers [60] very well.

## 3. Results and Discussion

**Figure 2:**
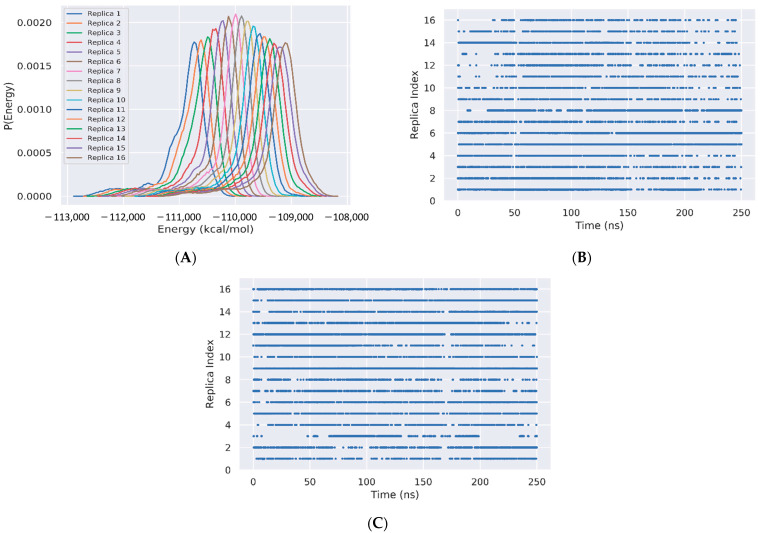
REST2 Sampling. (**A**) Overlap of the total potential energies for replicas 1 to 16. (**B**,**C**) Exchanges of coordinates as a function of simulation time of replica 1 and replica 16, respectively.

**Figure 3:**
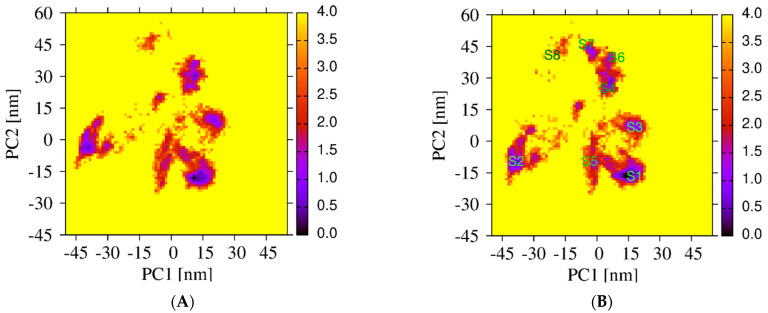

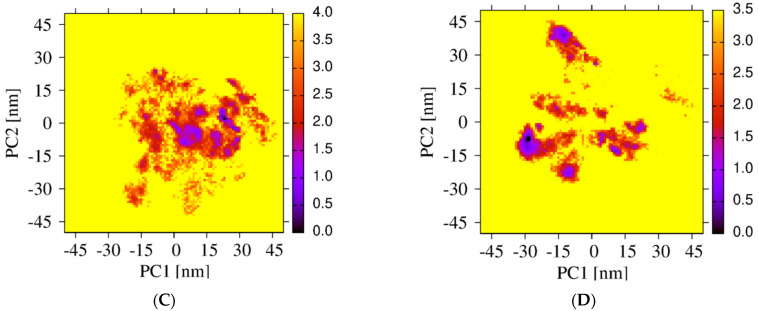
Free energy landscapes of Aβ42 trimer + 3 cholesterols from PCA. REST2 FEL by using the single REST2 trajectory at 310 K and time intervals 50–220 ns (**A**) and 50–250 ns (**B**). REMD FEL (**C**) and REST2 FEL (**D**) by using the combined REST2 (310 K) and REMD (315 K) trajectories and the time interval 50–250 ns. Note that the PC1 and PC2 differ from REMD to REST2.

The sampling of the REST2 simulation using 16 replicas is first illustrated by the good overlap of the distributions of the potential energy between neighboring replicas (Figure 2A). The exchanges of coordinates as a function of simulation time for replicas 1 (Figure 2B) and 16 (Figure 2C) indicate exploration of the full replica space in the nanosecond time scale. Overall, the average acceptance probability at different replicas is 0.3. The convergence of the REST2 simulation at 310 K is also assessed by the very high similarity of the two FELs using the single REST2 trajectory at the time intervals 50–220 ns and 50–250 ns (Figure 3A,B).

**Figure 4:**
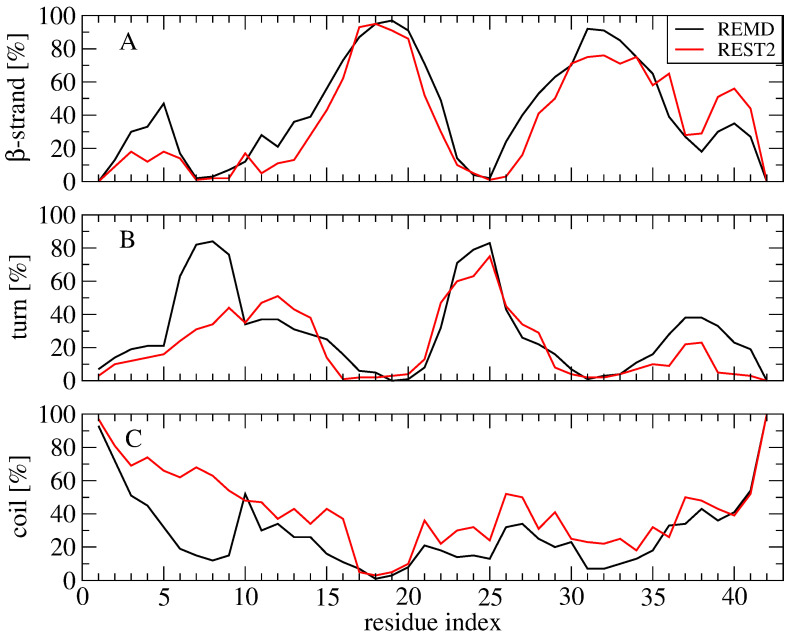
Secondary structure composition along the amino acid sequence using the time interval 50–250 ns of the REMD simulation at 315 K and the time interval 50–250 ns of the REST2 simulation at 310 K. (**A**) beta-strand, (**B**) turn and (**C**) coil content.

The secondary structure compositions of Aβ42 slightly vary between the two simulations, reaching β-sheet and coil contents of 37% and 36% in REMD vs. 36% and 43% in REST2. There are, however, differences along the amino acid sequence. Both methods give the same β-strand content for the residues 15–21 and 28–37 (Figure 4A) and the same turn probability for residues 22–27 (Figure 4B). The β-strand probability of residues 3–5 and the turn character of residues 6–10 are, however, reduced by a factor of two from REMD to REST2 (18% β-strand and 30% turn by REST2), leading to an enhancement of coil character of the N-terminus (residues 1–10) in REST2 compared to REMD (Figure 4C). Additionally, REST2 explores more β-strands at positions 36–41 (Figure 4A).

Differences in the conformational space explored by the REST2 and REMD simulations are further analyzed by comparing the two FEL’s using the combined REMD and REST2 trajectories to compute the first two principal components. Though the direct comparison is not possible because we use two distinct force fields, there is some overlap, but overall, the free energy landscapes are different. In contrast to the REMD FEL (Figure 3C), the REST2 FEL is divided into well-separated regions (Figure 3D). Additionally, the amplitudes of fluctuation along the PC1 and PC2 are also much larger in REST2, and the REST2 FEL is dominated by two states, S1 and S2, representing 40% and 22% of the full ensemble. In contrast, the REMD FEL is dominated by two states representing 12% and 28% of the conformational ensemble. 

**Figure 5:**
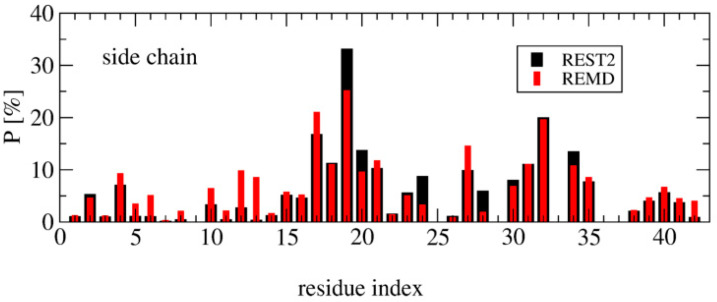
Probability of side-chain contacts between each cholesterol molecule and each amino acid of each Aβ42 peptide using the time interval 50–250 ns of REMD at 315 K, and REST2 at 310 K.

**Figure 6:**
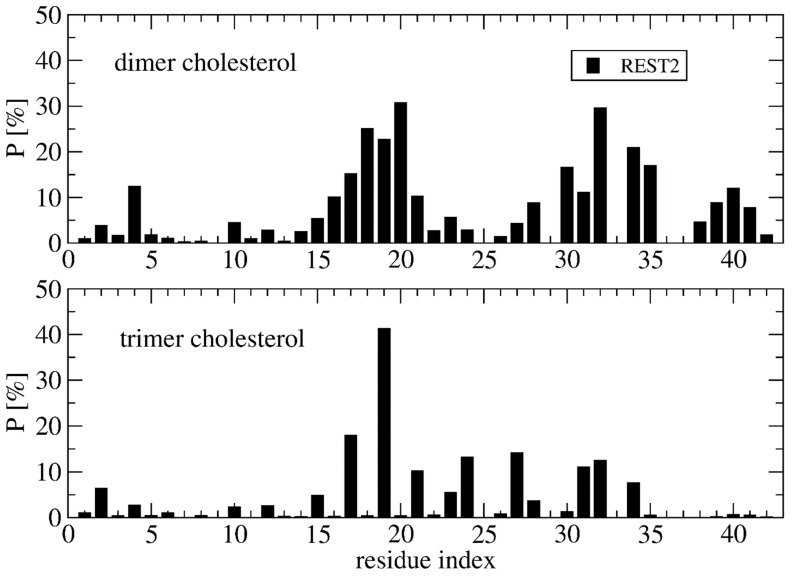
Probability of contact between the cholesterol molecules in dimer+monomer form and trimer form and the side-chains of Aβ42 using the time intervals 50–250 ns of REST2 at 310 K.

Deviation between the REST2 and REMD conformational ensembles is observed for the probability of side-chain contacts per cholesterol molecule and Aβ42 chain (Figure 5). Using REST2, the highest binding spot with cholesterol molecules involves the side-chains of the CHC region (residues 17–21) with a probability of 33% for the aromatic interaction with F19 and 17% for the interaction with L17, and then the side-chains of residues L30, I31 and I32 and residues L34 and M35 (average probability of 12%). The same residues are identified with REMD, but the probability decreases notably for F19 to 25% and increases moderately for the residues H6, Y10, V12, H13 and N27, with most probabilities remaining < 10%, however. There is almost no difference between the REMD and REST2 populations of (free monomers of cholesterol, dimers + monomer of cholesterol and trimers of cholesterol), as they reach (0.5%, 43% and 56%) in REST2 vs. (0.6%, 47% and 52%) in REMD. It is found that the network of interactions between the side-chains of Aβ42 varies with the aggregated forms of cholesterol. Using REST2, there are 15 contacts per Aβ42 chain and per cholesterol with a probability > 7.5% when cholesterol is in dimers + monomer form, while there are 8 contacts formed when cholesterol is in trimer form, and this is accompanied by a substantial probability reduction with the CHC region and residues 30–41 (Figure 6).

Overall, there are non-negligible differences between the REST2 and REMD results. REST2 results emphasize the role of the CHC region L17–A21 and the hydrophobic residues L30–M35 and V39–I41 in the binding with the dimer form of cholesterol, and the residues L17, F19, A21, V24, N27, I31 and I32 in the binding with the trimer forms of cholesterol. This binding mechanistic view between the hydrophobic region of Aβ42 and cholesterol explains why the nonvesicle forms of the negatively charged cholesterol sulfate and the cationic cholesterol derivative, 3β(N-dimethylaminoethane)carbaloyl)-cholesterol, moderately change the aggregation kinetics of Aβ40 [22]. Our cholesterol-binding mechanism with an average probability of contacts of 16% averaged over the residues 17–21 is clearly more complex than that previously described by short MD simulations, which only emphasized the critical role of the side-chain of F19 in the potentiation effect of cholesterol on Aβ40 fibril formation [24].

**Figure 7:**
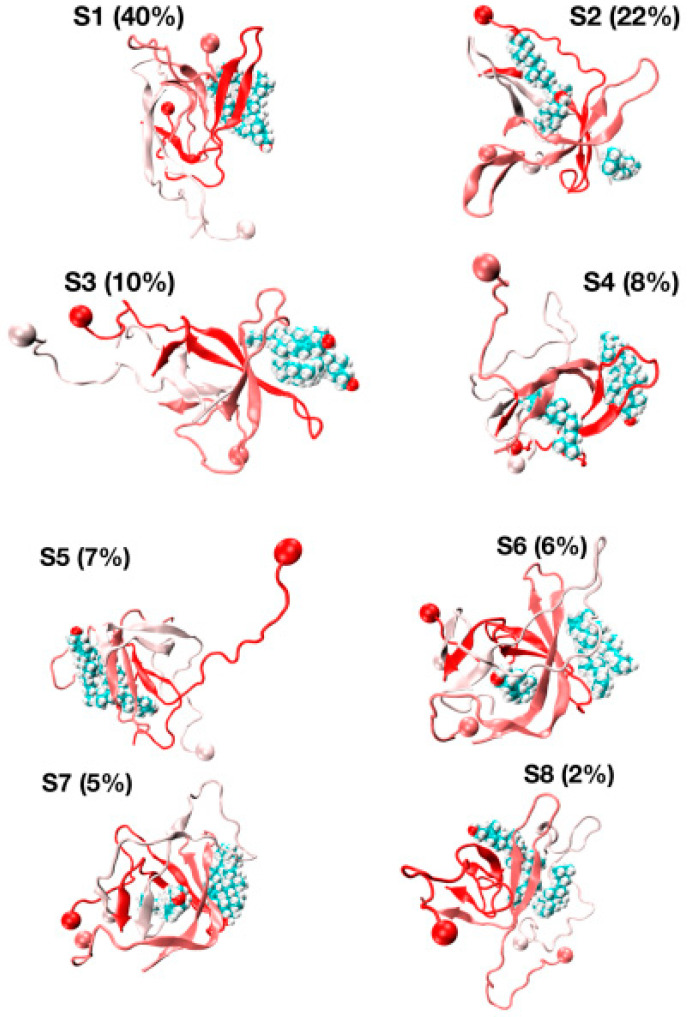
Representative structures and populations of eight free energy minima from REST2 simulation at 310 K. Chain A is in red, chain C is in white and chain B is in pastel pink. The ball shows the N-terminus of each chain. The cholesterols are visualized in all-atom and Van der Waals representations.

The representative structures of the eight free energy minima designated as S1–S8 on the FEL with decreasing populations are shown in Figure 7. The first minimum, S1, with a population of 40%, is characterized by three chains forming well-defined β-hairpins and chains A and B forming a short-twisted four-stranded antiparallel β-sheet. The β-strands cover residues L17–A21 and N27–I31 in chain A, residues L17–A21 and I32–V36 in chain B and residues H14–A21 and K28–M35 in chain C, indicating that the β-hairpins are distinct and extend beyond the CHC region by including some residues in the loop region (residues E22–K28). The second minimum (S2, population of 22%) displays two highly bent and twisted β-hairpins with strands covering residues L17–F20 and A30–I41 in chain A, residues L17–F20, K28–L34 and V39–I41 in chain C and chain B adopting a five stranded β-sheet at R5–S8, Y10–Q15, L17–E22, I31–V36 and V39–I41. The orientation of the three chains is complex as the C-terminus residues (30–41) of chain A are antiparallel with the K28–L34 residues of chain C, and the CHC of chain C is parallel to residues V39–I41 of chain C.

The S3, S4, S6 and S7 minima, with a total population of 29%, have a β-strand content of 36% and share the same topological features: two peptides with highly twisted β-hairpins (chains A and B) covering distinct amino acids perpendicular to each other, and a third disordered and compact peptide (chain C) with no preferred interface with the other two chains. For instance, there is a small intermolecular β-sheet between residues V40–I41 and K28–G38 in S3 and between residues L34–V36 and V39–I41 in S4. The S5 state with a population of 7%, dominated by a coil (49%) with low β-strand content (29%), forms a twisted four-stranded antiparallel β-sheet (chains A and B) with chain C having very deformed β-strands. Finally, the S8 state with a population of 2% is amorphous and has high turn and coil contents of 36% and 38%. S8 has a low β-strand content of 24% with strands at residues Q15–V18, L30–G33 and V40–I41 in chain A and residues V18–E22, I31–M35 and V39–V40 in chain B, forming a short intermolecular parallel β-sheet between the two termini of chains A and B. In this state, chain C is almost devoid of any secondary structure with the exception of a small helix covering residues A30–G33. 

**Figure 8:**
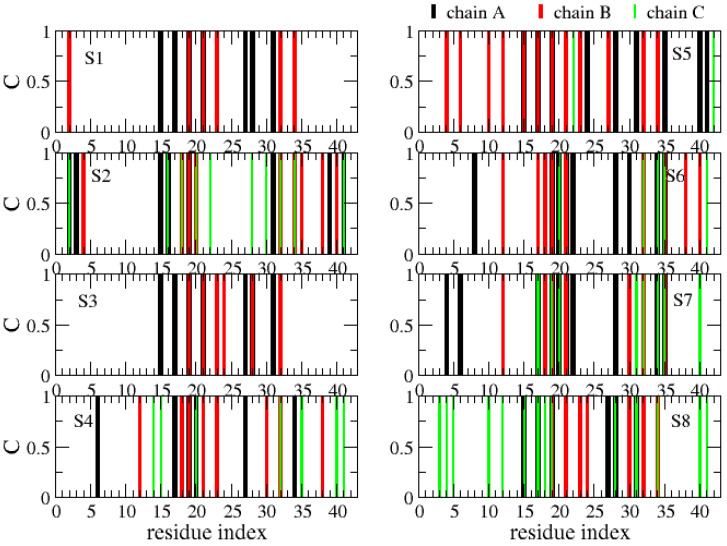
Contact between each Aβ42 chain (A, B, C) and cholesterols. Shown are results for each free energy minimum Si (i = 1…8). One Aβ42 side-chain and one cholesterol molecule are considered in contact if there is at least one intermolecular distance below 0.45 nm.

The various globular shapes of β-strand contents varying between 24% (S8 state) and 42% (S2) are all characterized by different orientations and packings of the chains, but also different binding interfaces and hot spots with cholesterol (Figure 8). In the S1 state, the residue hot spots with a trimer of cholesterol involve residues H14, K16, N27, K28 and I31 of chain A, and residues A2, F19, A21, D23, L30, I32 and L34 of chain B. In this state, cholesterol is located at the surface of the Aβ trimer (Figure 7, S1 state). The S3 state has almost the same residues for binding and a trimer of cholesterol located at the surface. The S5 state is also characterized by a trimer of cholesterol at the surface, but binding involves many residues in the F4–L34 region of chain B, the residues V24, K28, I31, I41 and A42 of chain A, and residues E22 and A42 of chain C (Figure 8). In total, 57% of Aβ states have a trimer of cholesterol at the surface. 

The S4, S6 and S7 states representing 22% of the ensemble are characterized by a dimer of cholesterol at the surface and one cholesterol inserted into the complex (Figure 7). In these states, we find that the residues 16–22 are essential for binding, but many residues from the region K28 to A42 and a few residues in the N-terminus (residues F4, H6, S8 and V12) participate as well (Figure 8). Finally, the S2 state reveals a dimer of cholesterol inserted in Aβ trimer and one cholesterol at the surface with binding residues D1–F4, Q15–V24 and K28–I41. In contrast, the S8 state reveals a trimer of cholesterol in the interior of oligomers with binding residues D1–Q15 and K16–V36 (Figure 7 panel S8 and Figure 8).

Overall, these results are very different from a previous MD simulation which only reported on interactions of Aβ42 peptides bound to the surface cholesterol micelle [22]. Our cholesterol-binding sites of Aβ42 also differ from combined docking modelling and surface pressure studies of Langmuir monolayers that suggested the region E22–M35 as the minimal cholesterol-binding site. It must be stressed that the docking procedure used the NMR structure of an Aβ monomer mixed with detergent micelles, characterized by an alpha-helix at residues Q15–V36 with a hinge at residues G25–N27 [61]. However, it is interesting that our simulations report on the contribution of the loop residues E22–K28 in the binding process.

**Figure 9:**
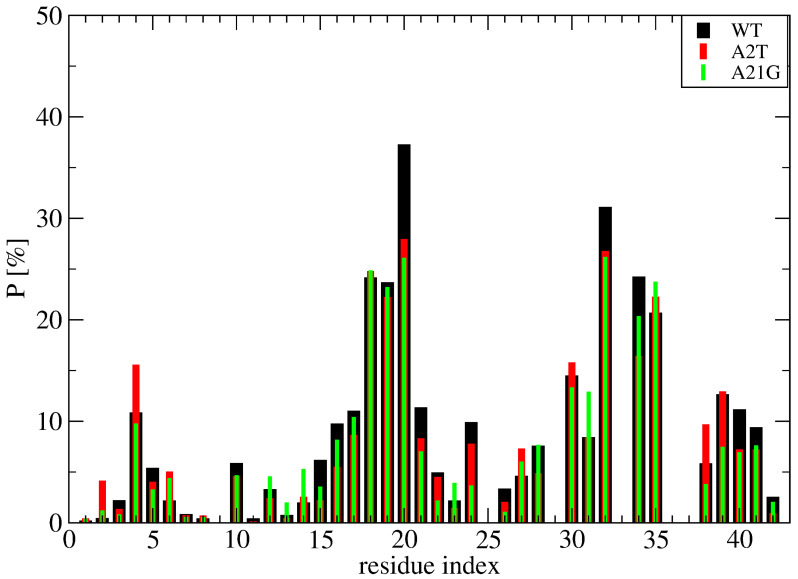
Time-averaged probability of contacts between the cholesterol molecules and the side-chain of Aβ42 obtained from 1 microsecond MD trajectory at 310 K of the wild-type (WT, black), A2T mutation (red) and A21G mutation (green) sequence. Shown are the averaged values over the three simulations starting from the S1, S2 and S4 structures displayed in Figure 7.

To further understand the binding mechanism of cholesterol, Figure 9 report the time-averaged probability of contacts between the cholesterol molecules and the side chains of wild-type (WT) Aβ42, A21G Aβ42 and A2T Aβ42 obtained from a total of 3 microseconds per system (namely 1 microsecond MD simulation starting from the S1, S2 and S4 structures at 310 K shown on Figure 7). Though the S1, S2 and S4 structures of WT represent 70% of the full conformation space of the wild-type sequence, they were selected because they display hot spots involving the N-terminus, the CHC, the loop region and the C-terminus. It is important to stress that these two mutations were also selected because A2T introduces a hydrophilic residue and thus potentially reduces the hydrophobic surface with cholesterol, and A21G because it reduces the total hydrophobic character of the CHC region. Our results show that both mutations do not change the profile of interactions per Aβ42 residue and per cholesterol in all regions of Aβ42, including the N-terminus and the CHC region (Figure 9). These results suggest that cholesterol should moderately alter the aggregation kinetics of Aβ42 A2T and Aβ42 A21G in the bulk solution, with an enhancement that should be comparable to that observed for WT Aβ42 [22].

## 4. Conclusions

In summary, we determined the Aβ42/cholesterol trimeric states by means of REST2 simulations and a force field designed for intrinsically disordered proteins. Consistent with our previous REMD simulation using a force field for well-structured proteins [30], we found that the conformational space does not contain the aggregation-prone state of the parallel U- and S-shape Aβ42 fibrils [2] but displays some antiparallel dimers with short intermolecular β-sheets built on strands located at different regions that go beyond the CHC region. Based on our previous REMD study of Aβ42/cholesterol dimers showing an increase of the population of β-hairpin and β-sheet contents upon cholesterol addition compared to a pure bulk solution [30], it is likely that all Aβ42 oligomers mixed with free cholesterol will show more β-structural and antiparallel β-sheet features of the peptides than in pure bulk solution as the oligomer size augments [50].

Our simulations also show that the formation of Aβ42 trimers in the presence of cholesterol involves two binding interfaces. A first highly populated one, where cholesterols are mainly located at the surface of Aβ oligomers (either as a trimer or a dimer of cholesterol), and a second much less populated where cholesterol is fully inserted in the interior of the Aβ oligomers. The predominance of the first binding interface implies that cholesterol should act as a glue to speed up the formation of larger aggregates and therefore catalyzes primary nucleation. This explains the modest but non-negligible experimentally observed acceleration of the aggregation rate of Aβ40 and Aβ42 in the presence of cholesterol [22].

Finally, we found that the binding hot spots of Aβ are very complex and go much beyond the CHC region (residues L17–A21) and the hydrophobic residues L30–M35. Our mechanism cannot be generalized to all amyloid proteins. Indeed, it was shown that free cholesterol has an inhibitory effect on the aggregation of the 37-residue amylin protein, which has two hydrophobic regions, LANFLV and FGAIL, separated by HSSNN, in both solutions and on model membranes [62]. Clearly, a better understanding of the interactions of free cholesterols or free phospholipids on amyloid aggregates in the brain either by computational [63] or experimental [25,64] means must be further explored. To this end, we are coupling a coarse-grained protein force field in an aqueous solution [65,66] with coarse-grained cholesterols and phospholipids [67] to explore larger aggregates and the impact of other disease-causing and disease-protecting mutations [68,69,70,71].

## Data Availability

Not applicable.
